# Feasibility and preliminary efficacy of remotely delivering cognitive training to people with schizophrenia using tablets

**DOI:** 10.1016/j.scog.2017.07.003

**Published:** 2017-08-03

**Authors:** Bruno Biagianti, Melissa Fisher, Lisa Howard, Abby Rowlands, Sophia Vinogradov, Joshua Woolley

**Affiliations:** a Department of Psychiatry, University of California, San Francisco, USA; b Posit Science, Inc., USA; c Department of Psychiatry, University of Minnesota, USA

**Keywords:** Schizophrenia, Cognitive remediation, Neuroplasticity, Mobile health

## Abstract

**Background:**

Limited access to Cognitive Training (CT) for people with schizophrenia (SZ) prevents widespread adoption of this intervention. Delivering CT remotely via tablets may increase accessibility, improve scheduling flexibility, and diminish patient burden.

**Methods:**

In this reanalysis of data from a larger trial of CT, we compared two samples of individuals with SZ who chose to complete 40 h of CT either on desktop computers in the laboratory (*N* = 33) or remotely via iPads (*N* = 41). We examined attrition rates and adherence to training, and investigated whether remote iPad-based CT and in-person desktop-based CT induced significantly different improvements in cognitive and real-world functioning.

**Results:**

The attrition rate was 36.6%. On average, participants completed 3.06 h of CT per week. There were no significant between-group differences in attrition and adherence to CT requirements. Participants who completed iPad-based CT were significantly younger and had lower symptoms at baseline compared to participants who completed CT on the lab desktops. Controlling for age and symptom severity, rANCOVA showed that iPad-based and desktop-based CT similarly and significantly improved verbal learning and problem solving. Main effects of time, at trend level significance, were evident in global cognition, verbal memory, quality of life, and social functioning. All group by time interactions were non-significant except for verbal memory, where iPad users showed greater gains. Within-group effect sizes for changes in outcomes were in the small range.

**Conclusion:**

Although underpowered and not randomized, this study demonstrates that delivering CT remotely to people with SZ using tablets is feasible and results in retention rates, adherence, and cognitive and functional outcome improvements that are comparable to those observed when CT is delivered in the laboratory. This has important implications in terms of scalability and dissemination of CT. These results require confirmation in larger samples.

## 1 Introduction

Schizophrenia (SZ) is associated with a wide range of Cognitive Impairments (CIs), including deficits in attention, speed of processing, learning and memory, problem solving, and executive functioning that are present early in the course of illness and are more enduring than psychotic symptoms (Green et al., 2004). These CIs undermine independent living and are associated with decreased lifelong community and occupational functioning even when psychotic symptoms are in remission (Kurtz et al., 2008). As a result, CIs account for 20–60% of the variance in functional outcome of individuals with SZ (Green, 1996). Discovering methods to treat CIs in SZ early in the course of illness, before functional and psychosocial deterioration has occurred, is a major goal of 21st century psychiatry. New treatment approaches to enhance cognition, both pharmacological and behavioral, have been tested for patients with SZ. To date, none of the pharmacological trials found significant effects on CIs compared to placebo (Keefe et al., 2013). However, there is growing evidence that an intensive computerized neuroplasticity-informed Cognitive Training (CT) program may be an effective treatment for CIs in SZ.

Our research group has demonstrated that CT targeting the auditory system in adults with SZ improves early dynamic imaging responses in auditory and prefrontal cortices, as well as global cognition, speed of processing, verbal learning, and verbal memory, and that such cognitive gains predict enhanced quality of life at 6 month follow-up (Biagianti et al., 2016a, Dale et al., 2010, Dale et al., 2015, Fisher et al., 2010, Fisher et al., 2016, Subramaniam et al., 2012). Additionally, a new set of exercises targeting processing speed and working memory in the social cognitive domain was tested in young adults with SZ. Results from the pilot study indicated significant improvements on prosody identification, facial memory, social functioning, motivation and reward sensitivity (Nahum et al., 2014). More recently, a randomized controlled trial found that supplementing CT with these social cognitive exercises in people with psychotic disorders confers greater benefits in prosody identification and reward processing relative to CT alone(Fisher et al., 2017). While preliminary evidence indicates that CT is efficacious, access to and engagement with CT by individuals with SZ remain outstanding challenges. This prevents widespread and optimal utilization of this promising intervention. In our studies, we have asked individuals with SZ to complete, without supervision, one hour of training exercises a day, five times a week, for a period of 8–10 weeks. Therefore, this intervention can place a high scheduling burden and become untenable for those who are in school or employed, have caregiver demands, or other medical appointments to manage. Additionally, CT is currently delivered as an experimental treatment in only a few specialized mental health clinics, and may not be accessible to people who live in rural or under-resourced areas or are without transportation (Kohn et al., 2004). The time spent reaching the clinic may therefore be another barrier to the implementation of this treatment. Lastly, some individuals with SZ hesitate to approach traditional mental health treatment settings because of stigma, which interferes with help-seeking behaviors (Angermeyer et al., 2013). These factors may all account for the high attrition rates found in CT trials, with important implications on the scalability of this intervention (Biagianti et al., 2016a).

Advances in interactive software development and health care delivery provide a unique opportunity to overcome these limitations. The rapid expansion of mobile technology in this population (Gay et al., 2016), with 81.4% of individuals with SZ owning a mobile phone (Firth et al., 2016), has already revolutionized the field of treatment development and delivery, allowing users to engage with innovative interventions, entirely remotely, anytime, anywhere, on their own schedule (Berry et al., 2016). Internet and mobile interventions are acceptable and feasible for individuals with SZ and have the potential to improve clinical and functional outcomes (Alvarez-Jimenez et al., 2014, Biagianti et al., 2016b). Similarly, the remote delivery of CT through mobile platforms enables scheduling flexibility and decreases scheduling burden, which may improve adherence to intervention requirements and ultimately increasing cost-effectiveness (Ventura et al., 2013). Additionally, through mobile platforms, CT can be delivered to individuals with SZ who are unable or unwilling to come in to the clinic. In doing so, mobile platforms may improve access, expand reach, and target underserved vulnerable populations.

Recent evidence suggests that CT can be feasibly delivered using mobile platforms. A 4-week feasibility trial delivering 20 h of iPad-assisted cognitive remediation vs. treatment as usual to 20 first-episode SZ in-patients showed significant improvements in working memory and good acceptability and adherence (Dang et al., 2014). More recently, our group demonstrated that it is feasible and acceptable to engage individuals with SZ in social cognition training entirely remotely using iPads (Biagianti et al., 2016b). Taken together, these findings indicate that delivering treatments including CT via internet and mobile platforms is acceptable and feasible. However, given the early state of current research, no studies have evaluated whether delivering evidence-based treatments like CT using online and mobile platforms is as efficacious as in-person delivery.

In this study, we analyzed data from a cohort of participants with SZ from our parent study (described below) who had a choice of completing 40 h of CT either on desktop computers in the laboratory, or remotely on iPads. We compared attrition rates and adherence to training requirements. We also investigated whether remote iPad-based CT and in-person desktop-based CT induce significantly different improvements in cognitive and real-world functioning.

## 2 Methods

### 2.1 Participants

This is a reanalysis of data from a double-blind Randomized Controlled Trial (RCT, ClinicalTrials.gov NCT02105779) that investigated the effects of supplementing CT with social cognitive exercises, as compared with CT alone (Fisher et al., 2017). From 2010 to February 2013, the CT program was only available on desktop computers in the laboratory. This changed in March 2013 when the CT program also became available on iPad devices that could be used remotely. From that point until the study's completion date (June 2016), participants were given the option to complete CT using desktop computers in the laboratory or to participate in the intervention remotely using provided iPads. There were no differences between the iPad and the desktop versions of CT in terms of the stimulus sets, stimulus progressions, adapting parameters, or logic of each exercise. The platforms only varied with respect to the user interface and exercise graphics.

The total number of participants randomized in the original trial was *N* = 111. In this study, however, post-hoc analyses were only performed on the subset (*N* = 74) of data collected from participants who enrolled in the study from March 2013 to June 2016, to allow for a more accurate comparison among participants who could choose both their device and location of use. This comparison is not based on the randomization criterion used in the parent RCT (CT + social cognitive exercises vs CT alone). Here, we directly contrast participants who freely chose to complete CT on iPads with those who chose to complete CT on desktops. Therefore, both groups include a mix of participants from the two treatment arms of the RCT.

Participants were recruited from the San Francisco VA Medical Center outpatient clinic, other local community mental health centers, and via presentations and online advertisements. Participants were clinically stable at the time of testing (no hospitalization and stable dosages of medication over the past month). Other inclusion criteria included: 1) fluent and proficient in English, 2) WTAR > 70 (Green et al., 2008), 3) sober during assessments and training, 4) no neurological disorder. Participants reported no prior cognitive remediation treatment. This study was approved by local Institutional Review Boards.

### 2.2 Procedures

All participants gave written informed consent for the study and were compensated for their participation in all assessments. Participants were asked to complete 40 h of the computerized CT program BrainHQ, provided free of charge by Posit Science Inc. After an intake evaluation that determined study eligibility, participants underwent an in-person structured diagnostic clinical interview and a battery of cognitive and clinical in-person assessments, which were administered again after training. For participants who completed CT in the laboratory, staff exposure during the intervention was kept to a minimum: staff aided all subjects to start each session but did not provide any coaching. Participants who opted for iPads were shown how to use the app BrainHQ in the lab before they returned home to engage in CT. Weekly phone calls were used to check in, monitor engagement, and offer technical support. Progress with CT was monitored remotely through the BrainHQ research portal. All participants received the same total number of hours of training and contact with research personnel. Multiple safeguards against loss of property were in place, including GPS tracking of the iPad and the option to remotely lock it, if lost or stolen. Security of electronic data was ensured at the level of the server, the devices, and the database.

All participants were compensated for their assessments and received $5 for each hour of CT and a $50 bonus at study completion. Payment was contingent on study participation but not performance. All patients did not have any identifiable motivation to feign or exaggerate symptoms. Patients were not using results of neuropsychological evaluations for disability compensation.

### 2.3 Cognitive Training (CT) program

The CT program consisted of auditory processing training exercises and auditory social cognition exercises that have been described in detail previously (Adcock et al., 2009, Nahum et al., 2014). In the auditory exercises, participants are driven to make progressively more accurate distinctions about the spectro-temporal fine-structure of auditory stimuli under conditions of increasing working memory load (i.e., increasing number of stimuli, and decreasing inter-stimulus intervals and duration of stimulus presentation). Stimuli across the exercises spanned the acoustic and organizational structure of speech, from very simple acoustic stimuli and tasks (e.g., time order judgments of rapidly successive frequency modulated sweeps) to the complex manipulations of continuous speech (e.g., narrative memory). Similarly, the social exercises harness the principles of brain plasticity, employing increasingly more challenging discriminations of socially-relevant stimuli, such as face identity, facial expressions, prosodic fluctuations, and gaze directions. Some exercises emphasize processing speed and require speeded discriminations, while others focus on memory, working memory, and attention load, which increase progressively during training. All exercises continuously adjust difficulty of the stimuli to an individual's performance, thus maintaining an 80%–85% correct performance rate in order to drive successful learning. We presented the auditory and social cognition modules serially over the course of the study, to avoid potential interference between the two modules. In each training session, a participant works with 4–6 exercises. Participants are asked to train for 60 min a day, 5 days a week. CT exercises are listed in Supplemental Table 1.

### 2.4 Measures

All neuropsychological assessments and clinical interviews were performed by highly trained raters, directly supervised by the same senior researcher (M.F.). Research staff who conducted clinical or cognitive testing first completed extensive training on testing/interviewing and scoring criteria of individual items (e.g., scoring videotaped sessions, observation of sessions conducted by experienced staff, and participating in mock sessions). Diagnostic assessments were administered at baseline, while all other assessments were administered at baseline and after 40 h of training.

#### 2.4.1 Diagnostic Assessment

At study entry, each participant received a standardized diagnostic evaluation performed by research personnel trained in research diagnostic techniques. Evaluations included the Structured Clinical Interview for DSM-IV Axis I Disorders (First et al., 2002), as well as review of clinical records and interview with patient informants (e.g., psychiatrists, therapists, social workers).

#### 2.4.2 Cognition

The MATRICS Consensus Cognitive Battery (MCCB; (Nuechterlein et al., 2008) was administered at baseline and at training conclusion. All primary outcome measures were distinct and independent from tasks practiced during CT. In addition to the learning trials, the verbal and visual memory trials (i.e., delayed recall) of the Hopkins Verbal Learning Test-R (HVLT-R) and Brief Visuospatial Memory Test-R (BVMT-R) were administered. Alternate forms of the HVLT-R, BVMT-R, and NAB Mazes tests were administered and counterbalanced. The following domains were assessed: attention, speed of processing, working memory, verbal learning and verbal memory, visual learning and visual memory, and problem solving. All tests were scored and rescored by a second staff member blind to the first scoring. The MCCB computerized scoring program was used to compute age and gender adjusted *T*-scores and the composite scores. *T*-scores for the HVLT-R and BVMT-R delayed recall trials were computed using normative data from the published manuals.

#### 2.4.3 Symptoms and functional outcome measures

To assess current (past 30 days) symptomatology, we used the Positive and Negative Syndrome Scale (PANSS, Kay et al., 1987). Higher scores are indicative of greater pathology. Quality of life was measured using the abbreviated Quality of Life Scale (aQLS, (Bilker et al., 2003). Social functioning was measured using the Social Functioning Scale (SFS, (Birchwood et al., 1990). Functional capacity was measured using the UCSD Performance Based Skill Assessment (UPSA, Patterson et al., 2001). Higher QLS, SFS, UPSA scores are indicative of better functioning.

### 2.5 Planned analyses

All variables were screened and normally distributed after Winsorizing of outlying values (± 2.5 SD from the mean). To investigate the feasibility and acceptability of remotely delivering CT to individuals with SZ using iPads, we compared attrition rates, hours of training completed, and training intensity between the two groups. Chi square was used to test for group differences in attrition rate. Independent sample *t*-tests were used to determine if there were any baseline differences between study completers with those who withdrew after baseline assessments. Fisher's Exact Test or Chi-Square Test tested for group differences in categorical variables (i.e., gender, diagnosis, medications, attrition). Bivariate correlations indexed the relationship between baseline characteristics and hours of training completed.

In order to determine whether the training delivery method (iPad vs desktop) had an effect on treatment response, we ran a per-protocol analysis on study completers only (*N* = 47). First, we examined differences in baseline demographic, cognitive, clinical, and functional characteristics, as well as hours of training completed and training intensity, between the two groups using Independent Samples T-tests. Repeated-measures analyses of variance were then used to compare the groups on the change in cognitive and functional outcome measures, controlling for potential baseline differences. Outcome scores collected at baseline and after training were entered as the repeated measure and training delivery method (iPad or Desktop) was entered as the between-subjects factor. Significant main effects of time accompanied by non-significant group by time interactions would suggest that subjects showed a significant change as a group, and that the training delivery method did not significantly influence this change. We also calculated change scores for outcome variables and explored bivariate correlations with demographics, hours of training, and training intensity.

Finally, in order to explore differences in the improvements induced by iPad-based vs. desktop-based CT, we first computed within-group effect sizes for each arm separately, by calculating the bias-adjusted standardized mean difference (Hedges' *g*), using the mean change scores (post treatment minus baseline) and the change score SDs. Next, we calculated confidence intervals for Hedges' *g* within-group effect sizes and conducted between-group comparisons to exclude statistically significant differences between desktop-based and iPad-based CT.

## 3 Results

Of the 74 participants who completed baseline assessments, 33 participants chose to complete 40 h of desktop-based CT condition in the laboratory, and 41 chose to be loaned iPads and complete the intervention remotely. 12 out of 33 (36.4%) subjects from the desktop-based CT condition withdrew from the study compared to 15 of 41 (36.6%) subjects from the iPad-based CT condition, a non-significant difference (×^2^ < 0.001, *p* = 0.984). The most common reason given for dropping out was finding the demands of the training too high and being unable to make time to do the training. There were no significant differences in demographic variables, medication use or dosage, cognition, symptom severity, or functioning between those who completed the study and those who discontinued (Supplemental Tables 2 and 3). These variables were also unrelated to the number of hours completed. Participants who discontinued had significantly fewer hours per week (*p* = 0.02) relative to study completers (see Supplemental Table 2). All iPads were returned undamaged and fully functional.

There were no significant differences in total hours of training completed and training intensity (hours of training completed per week) between those who completed the intervention remotely on iPads and those who completed it on desktop computers in the laboratory (Table 1). In particular, iPad users completed 2.84 ± 1.67 h of training per week, compared to 3.28 ± 1.28 h in the desktop-based CT group), which suggests good adherence to the training requirements.

**Table 1:** Characteristics of Participants who Completed Training via Desktop Computer and iPad.

	Completed training via desktop computer (*N* = 21) mean (SD)	Completed Training via iPad (*N* = 26) mean (SD)	T-test (*p*-value)
Female(N)/male(N)^a^	2/19	7/19	0.16
Age	49.24 (10.20)	40.38 (14.10)	2.50 (**0.02**)
Years of education	13.13 (2.39)	14.35 (2.51)	− 1.67 (0.10)
Wechsler test of adult reading-premorbid IQ estimate	99.52 (11.40)	104.60 (9.49)	− 1.5 (0.11)
Diagnosis^b^			
Schizophrenia(N)	14	17	
Schizoaffective disorder(N)	7	8	0.84(0.66
Psychosis NOS (N)	0	1	
Hours of training	40.00 (0.00)	41.86 (4.95)	− 1.88 (0.07)
Training intensity (hours/week)	3.28 (1.28)	2.84 (1.67)	1.00 (0.33)
Global cognition	27.86 (15.41)	31.18 (15.23)	− 0.73 (0.47)
Positive and Negative Syndrome Scale (PANSS) total	69.62 (15.58)	59.29 (14.23)	2.31 (**0.03**)
UPSA-brief^c^ total score	65.10 (15.46)	72.23 (14.74)	− 1.62 (0.11)
Quality of life scale-average item rating	2.62 (0.80)	3.20 (1.18)	− 1.92 (0.06)
Social functioning scale-average subscale total	104.63 (5.92)	108.14 (7.92)	− 1.65 (0.11)

Statistically significant p-values are formatted in bold.

a Fisher's Exact Test results.

b Chi-Square Test results.

c University of California, San Diego, Performance-Based Skills Assessment—Brief.

Participants who completed CT via iPad versus desktop computers were compared on demographic variables, baseline cognitive performance, symptoms, functional outcomes (Table 1) and medication regimens (Supplemental Table 3). All differences in baseline cognition, functioning and medication regimens were non-significant. Participants who completed iPad-based CT were significantly younger and had lower baseline symptoms compared to participants who completed CT on the lab desktops. Accordingly, repeated-measures ANCOVAs controlling for age and baseline symptom severity were used to compare the subject groups on the change in cognitive and functional outcome measures.

Omnibus test results showed significant main effects of time on Verbal Learning (*p* = 0.02) and Problem Solving (*p* = 0.05), and effects of time at trend level significance in Global Cognition (*p* = 0.07) and Verbal Memory (*p* = 0.06, Table 2). All group-by-time interactions were non-significant, except for Verbal Memory (*p* = 0.04). Post hoc analyses of Verbal Memory revealed non-significant improvements in the iPad participants (*F* = 0.45, *p* = 0.51), and a decrease at trend level significance in the desktop group (*F* = 3.47, *p* = 0.08). Interestingly, the effect size of the within-group change in Verbal Learning in desktop-based CT completers (*g* = 0.27, Table 3) was very similar to the one found in a sample of adults with persistent SZ who completed 50 h of CT on desktops (*g* = 0.29, Fisher et al., 2016), and to the one found among subjects of the parent trial who completed CT (*g* = 0.27, Fisher et al., 2017). Additionally, this effect size was similar to the one reported by a meta-analysis of existing cognitive remediation approaches (*g* = 0.39, McGurk et al., 2007). Conversely, the effect size for iPad-based CT completers (*g* = 0.13) closely resembles the within-group effect size found in a study conducted in recent onset SZ (*g* = 0.11, Fisher et al., 2015), where participants were loaned laptop computers and completed CT remotely and without supervision.

**Table 2:** Scores on Cognitive and Functional Outcome Measures at Baseline, and after 40 h of Training in Participants who Completed Cognitive Training (CT) in-person on Desktops (*n* = 21) or Remotely via iPads (*n* = 26).

	Desktop-based CT (*N* = 21)				iPad-based CT (*N* = 26)				Test statistics^e^	
	Baseline		After 40 h of CT		Baseline		After 40 h of CT		Main effects of time	Group by time interaction
									*p value*	*p value*
Outcome measures	Mean	SD	Mean	SD	Mean	SD	Mean	SD	F(p)	F(p)
Global cognition^a^	27.86	15.41	32.61	15.82	31.18	15.23	31.17	14.56	3.47 (0.07)	0.03 (0.88)
Attention^a^	36.10	14.65	36.53	14.40	39.78	12.45	41.79	15.84	0.12 (0.73)	0.26 (0.61)
Speed of processing^a^	33.14	14.41	34.32	18.00	34.62	13.70	36.56	14.82	0.96 (0.33)	0.11 (0.75)
Working memory^a^	36.19	14.13	37.29	14.83	42.06	14.59	41.62	11.17	0.08 (0.97)	0.52 (0.48)
Verbal learning^a^	34.10	6.83	36.52	10.65	38.54	11.42	39.88	8.51	6.08 (0.02)	0.85 (0.36)
Verbal memory^a^	29.36	18.21	23.86	17.30	29.66	18.00	31.83	18.48	3.84 (0.06)	4.77 (0.04)
Visual learning^a^	38.91	17.33	39.16	13.41	36.74	14.31	38.64	14.38	1.41 (0.24)	1.12 (0.30)
Visual memory^a^	39.59	18.16	34.50	17.55	36.98	18.55	36.66	16.38	1.20 (0.28)	1.17 (0.29)
Problem solving^a^	38.57	8.28	38.48	10.68	38.43	13.24	43.04	11.89	4.04 (0.05)	0.63 (0.43)
UPSA-brief^b^	65.10	15.46	65.50	17.45	72.23	14.74	74.30	14.15	0.59 (0.45)	0.00 (0.99)
Quality of life scale^c^	2.62	0.80	2.89	1.01	3.20	1.18	3.27	0.90	2.92 (0.10)	0.33 (0.57)
Social functioning scale^d^	104.63	5.92	106.92	7.04	108.14	7.92	109.19	7.14	3.62 (0.07)	0.43 (0.52)

a MATRICS Consensus Cognitive Battery (MCCB) Measures: Global Cognition (composite T-score across all MCCB measures); Attention (Continuous Performance Task-Identical Pairs); Speed of Processing (Trail Making Test Part A; Category Fluency Animal Naming; BACS Symbol Coding); Working Memory (Letter-Number Span; WMS-III Spatial Span); Verbal Learning (HVLT-R Immediate Recall); Visual Learning (BVMT-R Immediate Recall); Problem Solving (NAB Mazes). In addition to the MCCB, verbal and visual Delayed Recall from the HVLT-R and BVMT-R were administered.

b University of California, San Diego, Performance-Based Skills Assessment—Brief. Brief Total Score.

c Abbreviated Quality of Life Scale - Average Item Rating.

d Social Functioning Scale - Average Subscale Total.

e Repeated measures ANCOVA, controlling for age and PANSS symptoms, effects of time and group-by-time interaction.

**Table 3:** Effect Sizes and Confidence Intervals (CI) for the Within-group Changes in Cognitive and Functional Outcome Measures for Participants who Completed Cognitive Training (CT) in-person on Desktops (*n* = 21) or Remotely via iPads (*n* = 26).

	Desktop-based CT (*N* = 21)		iPad-based CT (*N* = 26)	
	Effect size		Effect size	
Outcome measures^a^	g^e^	95% CI	g^e^	95% CI
Global cognition^a^	0.30	(− 0.35; 0.95)	0.00	(− 0.57; 0.57)
Attention^a^	0.03	(− 0.61; 0.67)	0.14	(− 0.43; 0.71)
Speed of processing^a^	0.07	(− 0.57; 0.71)	0.13	(− 0.43; 0.69)
Working memory^a^	0.07	(− 0.55; 0.69)	− 0.03	(− 0.59; 0.52)
Verbal learning^a^	0.27	(− 0.36; 0.89)	0.13	(− 0.43; 0.69)
Verbal memory^a^	− 0.30	(− 0.93; 0.32)	0.12	(− 0.44; 0.67)
Visual learning^a^	0.02	(− 0.62; 0.65)	0.13	(− 0.43; 0.69)
Visual memory^a^	− 0.28	(− 0.92; 0.36)	− 0.02	(− 0.58; 0.54)
Problem solving^a^	− 0.01	(− 0.63; 0.61)	0.36	(− 0.2; 0.93)
UPSA-brief^b^	0.02	(− 0.6; 0.65)	0.14	(− 0.43; 0.71)
Quality of life scale^c^	0.29	(− 0.34; 0.92)	0.06	(− 0.53; 0.65)
Social functioning scale^d^	0.35	(− 0.29; 0.98)	0.14	(− 0.46; 0.74)

a MATRICS Consensus Cognitive Battery (MCCB) Measures: Global Cognition (composite T-score across all MCCB measures); Attention (Continuous Performance Task-Identical Pairs); Speed of Processing (Trail Making Test Part A; Category Fluency Animal Naming; BACS Symbol Coding); Working Memory (Letter-Number Span; WMS-III Spatial Span); Verbal Learning (HVLT-R Immediate Recall); Visual Learning (BVMT-R Immediate Recall); Problem Solving (NAB Mazes). In addition to the MCCB, verbal and visual Delayed Recall from the HVLT-R and BVMT-R were administered.

b University of California, San Diego, Performance-Based Skills Assessment—Brief. Brief Total Score.

c Abbreviated Quality of Life Scale - Average Item Rating.

d Social Functioning Scale - Average Subscale Total.

e Within-group bias-adjusted standardized mean difference (Hedges'g), calculated using the mean change scores (post treatment minus baseline) and the change score standard deviations.

Similarly, the effect size of the change in Problem Solving averaged across the two study groups (*g* = 0.18, Table 3) was similar to the one found in a sample of adults with persistent SZ who completed 50 h of CT on desktops (*g* = 0.12, Fisher et al., 2016), and to the one found among subjects of the parent trial who completed CT (*g* = 0.21, Fisher et al., 2017). However, this effect size was smaller than the one found within the group of individuals with recent onset SZ who completed 40 h of CT (*g* = 0.80, Fisher et al., 2015).

Omnibus test results showed main effects of time at trend level significance for the Quality of Life Scale (*p* = 0.10) and the Social Functioning Scale (*p* = 0.07), with both groups showing improvements over time (Table 2). The group-by-time interactions for these variables were non-significant. Hours of training and training intensity did not correlate with outcome change scores. For both groups, effect sizes of the within-group change in cognitive and functional outcome measures were in the small range (Table 3). Confidence intervals for effect sizes for all outcome measures overlapped between the two groups. This suggests that there were no statistically significant differences between changes induced by iPad-based CT and desktop-based CT.

## 4 Discussion

### 4.1 Feasibility and adherence

In this study, we examined the feasibility and preliminary efficacy of using tablets to remotely deliver 40 h of unsupervised computerized CT to individuals with SZ. We directly compared retention rates and adherence to the training between a sample of individuals with SZ who chose to complete CT using desktop computers in the laboratory and a sample of individuals with SZ who participated in the same intervention remotely using provided iPads. We found a non-significant between-group difference in attrition rates, suggesting that the training delivery method does not influence study retention. Additionally, we found no differences in pretreatment demographic, cognitive, clinical, and functional measures between study completers and dropouts. We also did not find baseline predictors of number of CT hours completed. Across groups, the most common reasons given for discontinuation were finding the demands of CT too high and being unable to make time to do the training. None of the iPad participants who withdrew from the study reported discomfort with using the tablet without supervision for CT.

Despite significant efforts to monitor, support, and keep participants engaged in CT, the overall study attrition rate was 36.5%. Similar attrition rates were found in the parent trial (Fisher et al., 2017)—where analyses on attrition were conducted after 70 h of training on all randomized participants— and in a study of CT in recent onset schizophrenia (Fisher et al., 2015). In these studies, some (Fisher et al., 2017) or all (Fisher et al., 2015) participants were loaned laptop computers or tablets with the necessary software and participated in the intervention remotely. However, the attrition rate found here is larger than the one found in our study of the same software in adults with persistent schizophrenia (15%, Fisher et al., 2016), where all participants performed the CT exercises in the laboratory. This suggests that delivering CT remotely via laptops or tablets may not necessarily improve study retention.

The average training intensity found in the group of iPad-based CT completers is in line with rates observed in other remote CT studies using similar versions of the program in young samples of individuals with SZ (Biagianti et al., 2016b, Nahum et al., 2014) as well as with the rates observed in our desktop CT group. We note that participants who chose to complete iPad-based CT were significantly younger and had lower baseline symptoms compared to participants who completed CT on the lab desktops. Participants who completed CT on iPads reported being comfortable using the device, liking the ability to schedule independently their training sessions and avoiding commute to engage in CT. Finally, all iPads were returned intact. Taken together, these findings indicate that delivering 40 h of CT remotely to people with SZ using tablets is feasible.

### 4.2 The effects of iPad vs. desktop training on cognition

The second goal of the study was to examine whether the improvements in cognitive and real-world functioning induced by iPad-based CT and desktop-based CT were different. Both groups showed significant improvements in verbal learning and problem solving, as well as improvements at trend level significance in global cognition and verbal memory. Additionally, the confidence intervals for the within-group effect sizes overlapped, indicating no statistically significant differences between improvements induced by iPad-based CT and desktop-based CT. Effect sizes for changes in outcome measures were in the small range. Inconsistent with previous reports (Fisher et al., 2015, Fisher et al., 2016), we found a decrease in verbal memory at trend level significance in desktop-based CT completers. This decrease, along with a non-significant improvement in the iPad participants, explains the significant group by time interaction observed for verbal memory. An intent-to-treat analysis of all randomized participants from the parent study showed significant gains in a greater number of cognitive domains (i.e. global cognition, attention, speed of processing, verbal learning, visual learning, and problem solving; Fisher et al., 2017). The lack of replication in this analysis of the subset is likely due to the smaller sample size, and the use of a per-protocol analysis.

### 4.3 The effects of iPad vs. desktop training on social functioning and quality of life

The whole sample also showed improvements in quality of life and social functioning at trend level significance. The CT delivery method (iPad vs desktop) did not influence the magnitude of these improvements. Within-group effect sizes for these improvements were in the small range. Overlap between the confidence intervals for these effect sizes indicates no statistically significant differences between iPad-based and desktop-based CT completers. Because the omnibus test of these functional outcome measures was at trend level significance, these results require confirmation with a larger sample size. It is also possible that improvements in functional outcome measures may be dependent on the domains targeted by CT, and may also not become evident immediately after the intervention. An open-label study that tested a set of exercises targeting processing speed and working memory in the social cognitive domain in young adults early in the course of SZ found significant improvements in social functioning immediately after training (Nahum et al., 2014). In previous imaging studies of adults with chronic SZ, we found that training-induced enhancement of prefrontal activity significantly predicted improvements in quality of life six months after completion of training (Subramaniam et al., 2014), and that gains in cognition were associated with gains in functioning, but only after six months (Fisher et al., 2010).

We note that the magnitude of the effects on both cognition and functional outcome measures in the present study was small, while in our prior studies, effect sizes ranged predominantly from medium to large (Fisher et al., 2015, Fisher et al., 2016). This difference is likely due to the fact that the computer games control condition used in our prior studies induced small, non-significant decreases in verbal cognitive measures, which contributed to the overall magnitude of the effects.

### 4.4 Limitations

The major limitation of this study is the lack of randomization. Since the CT program became available on iPads (March 2013), all participants enrolled in the study were given the option to choose their preferred training delivery method. This implies that any difference or lack of difference between the groups might be due to self-selection. We found that the two samples were unmatched for age and baseline symptoms. Although we controlled for these variables in our analyses of variance, the sampling bias limits the generalizability of the findings. Nonetheless, we believe that the quasi-experimental nature of this approach has revealed two important aspects regarding the acceptability of the mobile delivery method for CT in this population. First, 41 participants chose iPads to access CT, whereas only 33 opted for desktops in the laboratory. This provides evidence that acceptability is unlikely to represent a barrier to the implementation of CT on mobile platforms, and is in line with a recent review of delivered online and mobile interventions for serious mental illness, suggesting high acceptability particularly when participants are provided remote online support (Berry et al., 2016). Second, those participants who chose iPads for remote training were younger and less symptomatic, possibly reflecting a segment of individuals with SZ for whom tablets seem to be a preferable intervention delivery modality (Lal et al., 2015).

Another major limitation is the small sample size, which reduced our power to detect whether improvements in outcome measures were different or equivalent between the two groups. An intent-to-treat analysis (ITT) on all subjects could have identified additional between-group differences. However, when we conducted the ITT using a linear mixed-effects model with group and time as fixed factors, model convergence was not achieved for several outcome variables, thus compromising the validity of the models' fit. Additionally, the lack of significant between-groups differences demonstrated by this study does not imply statistically equivalent efficacy, i.e., that the magnitudes of improvements are significantly similar. Equivalence and/or non-inferiority testing methods, including the Two One-Sided Tests (TOST), require a larger sample size to statistically reject effects that fall outside the equivalence margins established for the effect size. Future studies of CT should be powered taking into account the attrition rates observed in this and other trials of unsupervised CT to draw conclusions about the comparative efficacy of these two training delivery methods.

We note that although the attrition rate found in this study is substantially higher than those found in other studies testing mobile interventions in individuals with SZ (Firth and Torous, 2015), CT – unlike most of these interventions – requires sustained effort, focused attention, and active planning and engagement by the user multiple days per week for several weeks. The high attrition rate may also be due to the fact that staff did not play an active role during the intervention, and that CT was not administered in the context of existing clinical services. These elements may explain the lower attrition rates found in similar studies of CT. For example, Fernandez-Gonzalo et al. found an attrition rate of 25% in outpatients with early illness schizophrenia who received individual coaching by neuropsychologists during all training sessions (Fernandez-Gonzalo et al., 2015). Lindenmayer et al. found an attrition rate of 7% when training was delivered to inpatients hospitalized for a 20-h per week rehabilitation protocol (Lindenmayer et al., 2013). Finally, the fact that similar retention rates were observed among those who completed the intervention on desktops in the laboratory and those who completed it remotely on tablets suggests that the high attrition is more attributable to the nature of the treatment rather than to its delivery method. Strategies that have been demonstrated efficacious at improving participation in other behavioral interventions that require ongoing commitment, like physical exercise for obesity, could help sustain engagement with CT. Examples include structured participation in groups rather than relying solely on at-home practice (Biagianti et al., 2016b, Hogarty and Flesher, 1999), behavioral economic approaches (Haff et al., 2015), motivational interviewing (Medalia and Saperstein, 2011), and gamification techniques to make the program more enjoyable (Lumsden et al., 2016). In addition to increasing study sample size, future studies should test and implement these strategies to sustain the engagement of study participants.

Other factors may limit the generalizability of the findings. First, long-term follow-up assessments are needed to determine the durability of treatment-induced gains and to evaluate whether improvements in functional outcome measures will become significant only after follow-up periods. Such studies are currently underway. Second, we did not place restrictions on medication regimens during study participation. Therefore, we cannot rule out non-specific effects of study participation and medication effects on the observed improvements. Third, the fact that participants were provided remuneration for each hour of CT completed likely biased data about engagement and adherence. Therefore, our results may not translate to real-world settings where this payment schedule may not be provided. Furthermore, research staff was not blind to the CT delivery method. Finally, participants in this study were well-educated, with an average age of 45, which may limit the generalizability of our results to other samples. For all these reasons, our findings should be taken with caution and replicated in future studies.

## 5 Conclusions

Our study suggests that remotely delivering CT to individuals with SZ using iPads is feasible, and results in retention rates and adherence to the training schedule that are comparable to those found when the same treatment is delivered in person in the laboratory using desktop computers. This has important implications in terms of access to and dissemination of CT. First, individuals with SZ living in under-resourced areas who are unable or unwilling to come in to the clinic could benefit from CT even if the treatment is not available locally. Second, through mobile platforms, providers will be able to monitor patient status remotely and provide support without requiring local infrastructures. It is important to note that the attrition rate found in this study –which seems independent of the delivery method – is likely to negatively impact treatment uptake in real-world settings. For this reason, additional research is needed to determine how to increase retention and sustain engagement. While preliminary in nature, our findings also indicate that the two training delivery methods are associated with similar improvements in verbal learning, global cognition, quality of life and social functioning, although these results require confirmation with a larger sample size.

In sum, we demonstrate here that the rapid expansion of mobile technology represents a major step forward in making CT readily available to individuals with SZ. We believe that the remote mobile approach used here to deliver CT can be successfully extended to other specialized treatment options. If so, remote digital technology will be an indispensable means to promote equity in access to mental health services and hopefully reduce disparities in mental health outcomes.

## Funding source

This work was supported by National Institute of Health NCT02105779.

## Appendix A Supplementary data

Supplementary Tables Image 1
