# Supplementary material for: Feasibility and preliminary efficacy of remotely delivering cognitive training to people with schizophrenia using tablets
Source: Schizophr Res Cogn. 2017 Aug 3;10:7–14. doi: 10.1016/j.scog.2017.07.003 (PMC5544490; doi:10.1016/j.scog.2017.07.003)
Supplement: Supplementary file 1 — Supplementary Tables [file mmc1.docx]

**Supplemental Table 1.** Targeted Cognitive Training Exercises Completed via Desktop and iPad.

| Desktop | | Description | | iPad | | Description | |
| --- | --- | --- | --- | --- | --- | --- | --- |
| AUDITORY | | | | | | | |
| High or Low | | Sound sweep processing: Indicate the direction of two consecutive sound sweeps. Each one can sweep either up or down in pitch | | **Sound Sweeps** | | Sound sweep processing: Indicate the direction of two consecutive sound sweeps. Each one can sweep either up or down in pitch | |
| Match It | | An auditory Memory game: Match pairs of sound cards in a memory game | | **Memory Grid** | | An auditory Memory game: Match pairs of sound cards in a memory game | |
| Tell Us Apart | | A syllable discrimination task: Select the syllable you just heard from a given pair. Syllables in the pair become increasingly similar as the difficulty increases | | **Fine Tuning** | | A syllable discrimination task: Select the syllable you just heard from a given pair. Syllables in the pair become increasingly similar as the difficulty increases | |
| Sound Replay | | Memorize the syllables you just heard and click on them in the order you heard them. The sequence becomes longer as the task progresses | | **Syllable Stacks** | | Memorize the syllables you just heard and click on them in the order you heard them. The sequence becomes longer as the task progresses | |
| Story Teller | | Answer questions regarding facts and memorized details in a segmented story | |  | |  | |
| Listen and Do | | An auditory WM task: Listen to a sequence of verbal instructions and perform the required tasks. The number of instructions increases as the task progresses | |  | |  | |
| SOCIAL (auditory) | | | | | | | |
| Prosody | | Correctly identify the prosody of the sentence with the neutral content | | **Voice Choice** | | Correctly identify the prosody of the sentence with the neutral content | |
| Face Prosody | | Match the prosody of a target sentence to the correct facial expression | | **Second that Intonation** | | Match pairs of vocal emotion cards based on their prosody in a memory task | |
| Prosody Memory | | Match pairs of vocal emotion cards based on their prosody in a memory task | | **Say What?** | | Select the correct vocal response given the situation described in the short audio script. | |
| Emotional Story Telling | | Answer questions regarding social interactions and facts in a segmented story | |  | |  | |
|  | | | | | | | |
| SOCIAL (visual) | | | | | | | |
| Face Match | | A speeded face matching task: Select the correct target face from an array of faces | | **Recognition** | | A speeded face matching task: Select the correct target face from an array of faces | |
| Gaze Match | | A speeded gaze matching task (match gaze direction of target face) | | **Gaze Match** | | A speeded gaze matching task: Match gaze direction of target face | |
| Mass Affect | | Select the visual scene that generates the same affect as the target scene, over gradually longer periods of time | | **Face to Face** | | A speeded facial emotion matching task: Select the face showing the same facial expression as the target face | |
| Name that Feeling | | A speeded facial emotion identification task, using still images: correctly name the emotion displayed on the target face | | **Face Facts** | | Memorize sequences of faces and personal facts on them. The sequence becomes adaptively longer given the user responses | |
| Second that Emotion | | Match pairs of facial expression cards based on their emotion in a memory task | | **Face Poke** | | A CPT task with facial expressions: Withhold response for neutral expressions, respond quickly to emotional faces | |

**Supplemental Table 2.** Characteristics of Participants who Remained and Dropped Out from the Study.

|  | Remained in the Study (N=47)  Mean (SD) | Dropped out from the Study (N=27)  Mean (SD) | T-test  (p-value) |
| --- | --- | --- | --- |
| Female(N) / Male(N)^a^ | 9/38 | 11/16 | (0.06) |
| Age | 44.34 (13.15) | 39.59 (14.50) | -1.44 (0.17) |
| Years of Education | 13.81 (2.51) | 13.81 (2.15) | 0.01 (0.99) |
| Wechsler Test of Adult Reading--Premorbid IQ Estimate | 102.28 (10.60) | 102.35 (14.13) | 0.02 (0.08) |
| Diagnosis^b^  Schizophrenia(N)  Schizoaffective Disorder(N)  Psychosis NOS | 31  15  1 | 15  11  1 | 0.66 |
| Global Cognition | 29.66 (15.23) | 29.83 (14.55) | 0.05 (0.96) |
| Positive and Negative Syndrome Scale (PANSS) Total | 64.11 (15.60) | 61.85 (17.86) | -0.56 (0.58) |
| UPSA-Brief^c^ Total Score | 69.04(15.32) | 67.70 (15.94) | -0.36 (0.72) |
| Quality of Life Scale ^d^ | 2.93 (1.05) | 3.47 (1.12) | 2.08 (0.06) |
| Social Functioning Scale ^e^ | 106.47 (7.18) | 108.43 (10.11) | .95 (0.35) |
| Dose (hours of training) | 41.01 (3.73) | 10.71 (10.65) | -14.03 (**<.001**) |
| Training Intensity (hours/week) | 3.04 (1.50) | 2.06 (1.70) | -2.51 (**0.02**) |

^a^ Fisher’s Exact Test results;

^b^Chi-Square Test results;

^c^ University of California, San Diego, Performance-Based Skills Assessment—Brief;

^d^ Abbreviated Quality of Life Scale -- Average Item Rating;

^e^ Social Functioning Scale -- Average Subscale Total.

**Supplemental Table 3.** Medication Regimens of Study Completers.

|  | **Completed Training via Desktop Computer (N=21)**  **Mean (SD)** | **Completed Training via iPad**  **(N=26)**  **Mean (SD)** | **Total**  **(N=47)**  **Mean (SD)** | **Fisher’s Exact Test p Value** |
| --- | --- | --- | --- | --- |
| **Antipsychotic Medication^a^** | | | | |
| 1^st^ Generation (N) | 2 | 6 | 8 | 0.27 |
| 2^nd^ Generation (N) | 16 | 19 | 35 | 1.00 |
| Multiple (N) | 2 | 2 | 4 | 1.00 |
| No Antipsychotic (N) | 4 | 2 | 6 | .39 |
| **Other Psychiatric Medication** | | | | |
| Antidepressants or Mood  Stabilizers (N) | 11 | 15 | 26 | 0.77 |
| Benzodiazepines (N) | 2 | 4 | 6 | 0.68 |
| **Other Medication Measures**^b^ **T-test (p value)** | | | | |
| Cogentin Equivalents | 0.57 (1.40) | 0.95 (1.85) | -- | -0.69 (0.50) |
| Chlorpromazine Equivalents | 325.45 (165.42) | 243.80 (180.86) | -- | 1.25 (0.22) |

^a^First generation antipsychotic medication = halperidol, perphenazine, thiothixene, trifluoperazine;

Second generation antipsychotic medication = aripiprazole, clozapine, olanzapine, quetiapine, risperidone, ziprasidone.

^b^ Mean and SD of Cogentin and Chlorpromazine Equivalents (Andreasen et al., 2010).
